# Impact of distress and anxiety due to COVID-19 on digital addictions in university students in the third wave period 

**DOI:** 10.12688/f1000research.154696.2

**Published:** 2025-01-27

**Authors:** Walter Capa-Luque, Luz Elizabeth Mayorga-Falcón, Evelyn Barboza-Navarro, Armando Martínez-Portillo, Yovana Pardavé-Livia, Edmundo Hervias-Guerra, Aldo Bazán-Ramírez, Catalina Bello-Vidal

**Affiliations:** 1 Facultad de Psicología, Universidad Nacional Federico Villarreal, San Miguel, Lima Región, Peru; 2 Escuela de Psicología, Universidad Nacional Jose María Arguedas, Andahuaylas, Apurimac, Peru; 3 Facultad de Medicina, Universidad Nacional Federico Villarreal, San Miguel, Lima Región, Peru

**Keywords:** Digital addictions, addictive behaviors, anxiety, distress, mental health, smartphone, internet, video games

## Abstract

**Abstract:**

**Background:**

Digital addictions are a major problem worldwide, which has increased considerably during the COVID-19 pandemic. In this scenario, two important impact factors to explain this problem are stress and anxiety because of COVID-19. The objective of this research was to determine the impact of distress and anxiety due to COVID-19 on digital addictions.

**Methods:**

cross-sectional, explanatory study. A total of 802 students from public and private universities residing in the city of Lima and Callao (Peru), with a mean age of 21.68 (SD = 3.11), selected by convenience sampling, participated in the study. The MULTICAGE CAD-4 questionnaire, the distress scale, and the anxiety scale by COVID-19 were applied.

**Results:**

two models examined with structural equation modeling showed good fit indices (CFI and TLI > .95, RMSEA and SRMR < .06). The first model shows that the latent variables distress and anxiety due to COVID-19 have direct effects on digital addictions as a general construct (R ^2^ = 22%). The second model shows that the exogenous latent variables (stress and anxiety) have direct effects of different magnitudes on each digital technology, so the variance explained on smartphone addiction was higher (R ^2^ = 25%) with respect to internet (R ^2^ = 19%) and video game addiction (R ^2^ = 6%). It was also found that for every male, there are two females with high levels of distress and anxiety. Regarding the problematic use of smartphones and internet, there is a prevalence of 40% regardless of sex; but as for the problematic use of video games, there is a marked difference between males (18.8%) and females (2.7%).

**Conclusion:**

the distress and anxiety caused by COVID-19 have a direct impact in aggravating digital addictions.

## Introduction

In the present century, the COVID-19 pandemic is classified as the most serious because of its person-to-person transmission, with asymptomatic carriers and its high magnitude of contagion ( Zhao et al., 2020). Globally, the COVID-19 pandemic has spread since December 2019 with consequences of risk of death due to viral infection and with many adverse psychological effects on the population ( Bao et al., 2020). Because of the coronavirus, the general population has experienced psychological problems such as anxiety, depression and stress ( Huarcaya-Victoria, 2020). According to the systematic review by Xiong et al. (2020), symptoms of depression, anxiety, psychological distress, stress, and post-traumatic stress disorder have increased markedly.

The university population is at risk for exposure to stress and anxiety ( Deng et al., 2021; Saladino et al., 2020). Studies highlight that the university population has been affected by the pandemic. For example, 24.9% of Chinese university students presented with anxiety due to COVID-19 ( Cao et al., 2020). University students in Bangladesh who were affected by the pandemic presented with anxiety between moderate (48.41%) and severe (44.59%) levels ( Dhar et al., 2020). In Iran, the overall prevalence of anxiety was 33.2%, depression 26.1% and stress 5.8% ( Parvar et al., 2022). In Jordan 70.6% of students reported mild to severe levels of anxiety. And those who were afraid of catching COVID-19 presented higher anxiety ( Masha'al et al., 2022). Also, Savitsky et al. (2020) found in a sample of Israeli university students the presence of moderate (42.8%) and severe (13.1%) anxiety. For their part, Rudenstine et al. (2020) reported that 20.7% of New York college students had severe anxiety during the COVID-19 pandemic. Likewise, Biber et al. (2020) found in a sample of U.S. college students that 49% had moderate anxiety and 25% had severe anxiety. In Peru, Sandoval et al. (2023) found in medical students’ levels of depression (24.3%), anxiety (28.5%) and stress (13.0%) in the categories of moderate, severe, or extremely severe.

In addition, the COVID-19 pandemic not only brought with it a decrease in person-to-person social interactions, but also an increase in the excessive use of the internet, becoming another critical problem which has extended the time spent in front of screens and increased digital connection ( Ferrara et al., 2021). This has modified leisure patterns and use of cell phones with an increase in online hobbies, such as games and video games ( Lemenager et al., 2021). In the meta-analysis by Meng et al. (2022), the prevalence of addiction to cell phones is reported at 26.99%, to social networks at 17.42%, internet at 8.23% and to video games at 6.04%; they also observed a pattern of increase in these variables over the last decades, with a significantly higher rate during the COVID-19 pandemic. Sandoval et al. (2020) found that 31% of medical students at the Ricardo Palma University (Peru) were addicted to the internet, and those who failed more courses had more problems with the internet; they also found that males had more problems with video games. Similarly, another study with students at the University of Malaga revealed excessive and problematic use of video games, as well as the use of cell phones was placed in the categories of risk and abuse ( Ruiz-Palmero et al., 2021).

In the academic environment, the mandatory implementation of virtual classes at the university generated changes in the lifestyle of students. The pandemic and the characteristics of virtual classes implied a considerable amount of academic work and online evaluations when they did not have the technological means or the skills to develop them effectively, pushing the students' adaptive capacity to the limit, causing a considerable number of students to present psychological problems such as stress, anxiety, intolerance to uncertainty, depression, as well as various physical health problems ( Bazán-Ramírez et al., 2022; Capa-Luque et al., 2023).

### Conceptual precisions for the variables addressed in this research

**Distress**. Der Feltz-Cornelis et al. (2020) indicate that distress is the negative stress that originates when the stressor stimulus and its exposure time increase and is characterized by anticipated negative thoughts about a particular event, anxiety, exhaustion, discomfort, restlessness, maladaptive states, emotional responses such as annoyance, sadness, phobias, depression, and psychoticism, among others.

**Anxiety due to COVID-19.** Anxiety is an excessive emotional reaction to circumstances that are intuitively recognized as threatening, and this emotion may be accompanied by agitation, exhaustion, muscular rigidity, and attention deficit ( Parvar et al., 2022).

**Digital addictions**. The World Health Organization - WHO (2022) divides the phenomenon of addiction into those originated by the consumption of a substance and those that are due to behaviors in themselves. Addictive behaviors are considered problematic because of the lack of control by the individual, as well as the maintenance of this behavior despite having multiple negative repercussions in his or her life ( Carbonell et al., 2021).

Internet addiction is conceived as a particular type of digital addiction ( Sarıalioğlu et al., 2021). It would be defined as lack of control over internet use ( Lozano-Blasco et al., 2022), involving compulsive behavior in terms of use ( Haberlin and Atkin, 2022), which can result in mood changes, tolerance, withdrawal, conflict, and relapse ( Young et al., 2011). During the COVID-19 pandemic, the likelihood of internet addiction would have increased significantly due to increased time of use ( Winther and Byrne, 2020).

Cell phone addiction is also recognized under other names such as problematic use, overuse, dependence, or smartphone addiction ( Ran et al., 2022). It is defined as a behavioral addiction with negative effects on psychological, social, and physical functioning caused by excessive or abusive cell phone use ( Peng et al., 2022; Ran et al., 2022), characterized by obsessive thoughts about the device, tolerance and elevated anxiety to cell phone withdrawal ( Peng et al., 2022).

Griffiths (1995) refers that video games are among the technological addictions and defines it as a behavioral addiction with compulsive involvement, lack of interest in other activities, and excessive human-machine interaction, which meets the criteria of prominence, mood change, tolerance, withdrawal symptoms, conflicts and relapses.

Generally, the studies that have been developed so far report the unfavorable psychological consequences of technological addictions and their impact on the performance and development of students; however, there is little research that explains the psychological causes that aggravate or favor students to develop technological addictions.

### Purposes of this research

According to the data found in publications from 2020 to 2022, a common pattern reported in international and national journal articles was the increase of addictive behaviors or problematic use of technological devices, as well as the increase of mental health problems in the university population (in particular the exacerbation of negative emotions). In this scenario, we did not properly find a psychological model that would enable an explanation of the increase in digital addictions.

**Figure 1.**
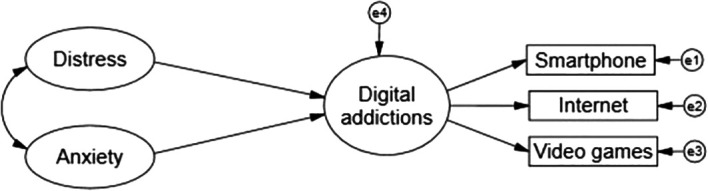
Theoretical explanatory model.

The present study emerged as a project between January and February 2022, when the third wave of COVID-19 infection was beginning to grow rapidly in Peru. After the traumatic experience lived during the first and second waves, as well as the measures adopted by the government to counteract the contagion through social isolation, mandatory quarantine, virtual classes in universities, in addition to the accumulated research that showed the high prevalence or increase of negative emotions and the increase in the excessive or problematic use of various technologies, the following hypothesis emerged: distress and anxiety due to COVID-19 are factors with direct impact and aggravate the problematic use of digital and interactive devices with the consequent resultant propitiating pictures of digital addictions in Peruvian university students (see Figure 1).

If the proposed model is empirically validated, it would be very useful to propose new intervention modalities or strategies to counteract or reduce the problems associated with the excessive or addictive use of interactive technologies such as smartphones, internet and video games. As Zhang et al. (2020) argue, using a new explanatory model may allow the design or planning of effective interventions in the context of health problems such as the Covid-19 pandemic.

In the context of the analyzed, the main objective of the research was to determine the impact of distress and anxiety due to COVID-19 on digital addictions in Peruvian university students in the third wave period.

## Methods

### Research design

The study corresponds to a non-experimental, cross-sectional, explanatory design ( Ato et al., 2013). Due to the nature of the analysis of relationships configured by latent variables, according to Byrne (2010) it corresponds to structural equation modelling.

### Participants

The population consisted of university students of different professional careers, residents of metropolitan Lima and Callao (Peru), enrolled during the first semester of 2022 in public and private universities, of both sexes, between 18 and 35 years of age. A total of 802 students participated in the study. Sampling was non-probabilistic by convenience. The sample was mostly female (64.1%), single (96%), students of health sciences (57.4%), from a national university (62%), about 50% were infected with COVID-19, with a family member infected in most households (69%), a third with a deceased family member in the household, and most had the third dose of vaccine against COVID-19 (84.4%).

### Instruments

**MULTICAGE CAD-4 Questionnaire.** This questionnaire was designed by Pedrero-Pérez et al. (2007). From the version adapted by Rodríguez-Monje et al. (2019) that assesses behavioral addictions, three of the scales were taken for the present study: internet abuse (items 17 to 20), video games (items 5 to 8) and problematic cell phone use (items 13 to 16). Each scale comprises of four items with dichotomous response (yes = 1, no = 0). The terms CAGE and CAD are acronyms that accompany the term multiple which alludes to a multi-scale instrument. Each scale (CAGE) has four items because it evaluates self-perception of the problem, perception by cohabitants, associated feelings of guilt and signs of abstinence or inability to control the behavior. Satisfactory validity evidence is reported based on the internal structure of the construct (GFI = 0.99, AGFI = 0.98, RMR = 0.002) and Cronbach's alpha values greater than 0.85 for all self-report scales ( Rodríguez-Monje et al., 2019).

In the present study for the three factors, very satisfactory fit indices were obtained that support the validity based on the internal structure of the construct (χ ^2^ = 228.631, df = 50, p = 0.000; CFI = 0.97; TLI = 0.96; RMSEA = 0.06, SRMR = 0.08); the estimated internal consistency with McDonald omega was 0.77, 0.80 and 0.93 for smartphone, internet and video game use, respectively.

**Distress Scale (ED).** A unidimensional self-report designed by Sandín et al. (2020) was used to assess distress based on items with indicators related to negative emotional experiences. It consists of 11 items with a 5-anchor Likert response format ranging from never (1) to always (5). The authors report high reliability estimated by internal consistency (α = 0.93, ɷ = 0.93). For use, validity (χ ^2^ = 341.884, df = 35, p = 0.000; CFI = 0.98; TLI = 0.98; RMSEA = 0.05, SRMR = 0.04) and reliability (omega = 0.93). The scale scores were estimated very satisfactorily in this study.

**Anxiety Scale by COVID-19.** To the scale designed by Matta et al. (2022) to assess COVID-19 anxiety, which consists of three items (I am worried about COVID-19, I am afraid of getting COVID-19 or becoming infected again, I am afraid of facing a situation related to COVID-19), an item “I am afraid of losing my life because of COVID-19” taken from the Coronavirus-19 fear scale by Ahorsu et al. (2020) was added. It presents response options ranging from strongly disagree (1) to strongly agree (7).

For its use in the present study, the psychometric properties of validity and reliability were examined. The confirmatory factor analysis fit indices estimated with WLSMV (χ ^2^ (2) = 42.885, p = 0.000; CFI = 0.998, TLI = 0.998, RMSEA = 0.038, SRMR = 0.017) confirm the validity based on the internal structure of the construct. The internal consistency estimated with ordinal alpha (0.94) and McDonald's omega (0.94) evidence high reliability for the instrument scores.

### Procedures

The instruments were administered through a format designed in Google Drive, for which the URL link was sent to the target population via email, WhatsApp, and Facebook. Only the students who offered consent were able to reply to the instruments anonymously.

Prior to data transformation for analysis, data quality control was performed, eliminating cases with zero variance and cases with extreme data according to the multivariate centroid analysis with the Mahalanobis distance, and then data processing was performed according to the research objectives. The descriptive analysis of the variables was executed with frequency analysis; the latent structure regression models were examined with structural equation modeling. The statistical programs used according to the requirements were jamovi version 2.3.28 ( The jamovi project, 2024), the free distribution package R version 4.3.1 ( R Development Core Team, 2020) and RStudio version 2023.06.2 ( RStudio Team, 2023).

## Results

### Levels of negative emotions (distress and anxiety due to COVID-19) in university students

Table 1 shows two females for every male with a high level of distress. Likewise, a high level of anxiety due to COVID-19 is observed in approximately 3 out of 10 females and 2 out of 10 males.

**Table 1.** Negative emotions in university students.

	General sample n = 802	Females n = 514	Males n = 288
	n (%)	n (%)	n (%)
**Level of distress**			
Absence	9 (1.1)	5 (1.0)	4 (1.4)
Low	134 (16.7)	80 (15.6)	54 (18.8)
Moderate	540 (67.3)	336 (65.4)	204 (70.8)
High	119 (14.8)	93 (18.1)	26 (9.0)
**Level of anxiety**			
Light	211 (26.3)	117 (22.8)	94 (32.6)
Moderate	398 (49.6)	257 (50.0)	141 (49.0)
Strong	193 (24.1)	140 (27.2)	53 (18.4)

### Descriptive levels of digital addiction risk in college students

Table 2 shows that around 4 out of 10 have problems with smartphone use and one third have problems with internet use, regardless of gender. Likewise, for every woman there are 2.6 times more men with problems in the use of video games.

**Table 2.** Percentage analysis on the use of interactive digital technologies.

	General sample n = 802	Females n = 514	Males n = 288
	n (%)	n (%)	n (%)
Smartphone Use			
Without problems	286 (35.7)	183 (35.6)	103 (35.8)
Occasional problems	202 (25.2)	122 (23.7)	80 (27.8)
With problems (addiction risk)	314 (39.2)	209 (40.7)	105 (36.5)
Internet Use			
Without problems	387 (48.3)	267 (51.9)	120 (41.7)
Occasional problems	156 (19.5)	93 (18.1)	63 (21.9)
With problems (addiction risk)	259 (32.3)	154 (30.0)	105 (36.5)
Video game Use			
Without problems	683 (85.2)	479 (93.2)	204 (70.8)
Occasional problems	51 (6.4)	21 (4.1)	30 (10.4)
With problems (addiction risk)	68 (8.5)	14 (2.7)	54 (18.8)

### Structural relationships between distress and anxiety by COVID-19 on digital addictions

The proposed theoretical model is supported by the empirical evidence because the goodness-of-fit indices turn out to be very favorable: χ ^2^ (132) = 525.061, p = 0.000; CFI = 0.979; TLI = 0.975; RMSEA = 0.061 [0.056, 0.066]; SRMR = 0.044; that is, the robust SEM model presents very good incremental fits (CFI and TLI) by being greater than 0.95 ( Hu and Bentler, 1999), and the absolute fit index RMSEA that evaluates the closeness to a perfect model denotes adequate fit because it presents a value less than 0.08 and the SRMR index that evaluates the error in the model reproduction by presenting value less than 0.06 indicate a very good fit ( Hu and Bentler, 1999).

**Figure 2.**
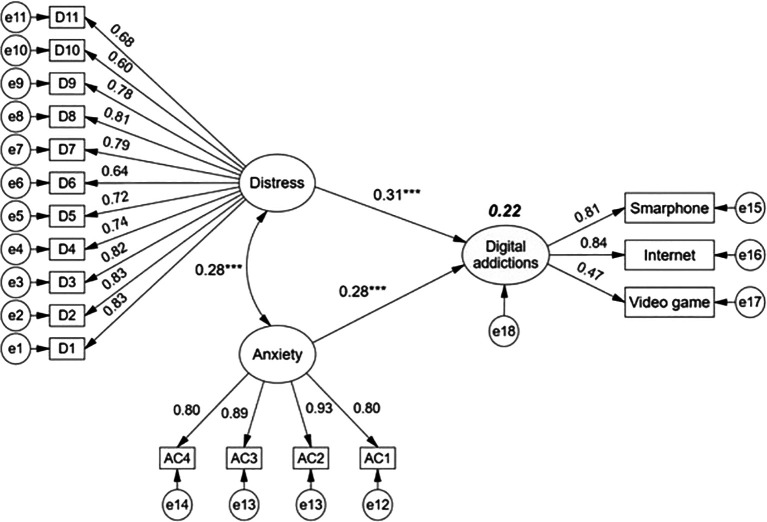
Structural regression model with effects of distress and anxiety on digital addictions.

The structural model ( Figure 2) allows us to observe that the latent variables distress and anxiety due to COVID-19 have direct and positive effects on digital addictions, which as a latent construct is configured by addiction to smartphone, internet and video games. Path coefficients evidence that distress (β = 0.31, p < 0.001) is of higher incidence than anxiety due to COVID-19 (β = 0.28, p < 0.001). The joint impact of the two exogenous variables on digital addictions in college students is 22%. On the other hand, the exogenous variables present a positive covariance of low magnitude, denoting absence of collinearity or overlap.

**Figure 3.**
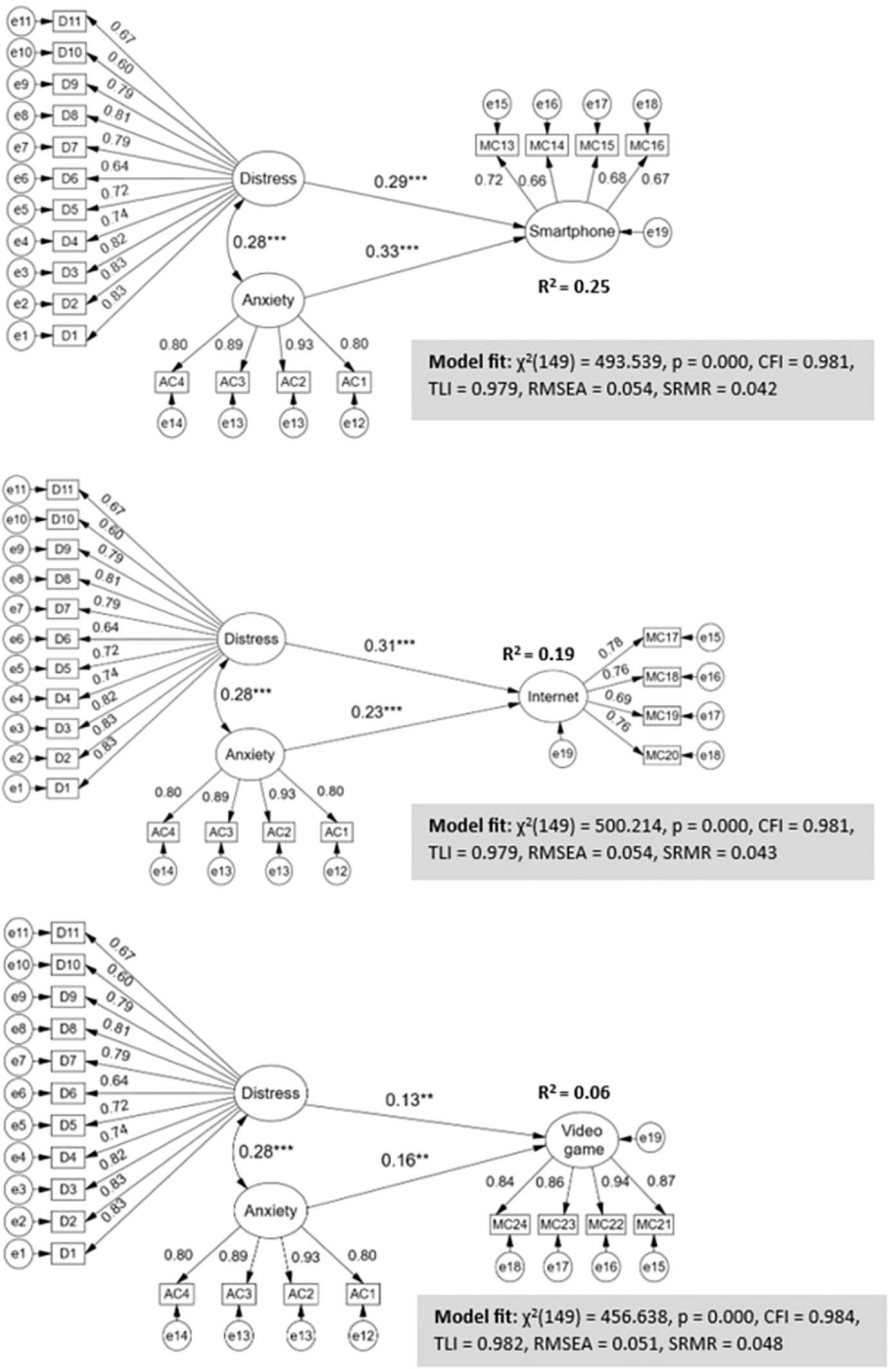
Structural regression model with effects of distress and anxiety by CODID-19 on digital addictions.

In the specific proposed models evaluate the effects of distress and anxiety due to COVID-19 on each of the interactive technologies with addictive potential such as smartphones, internet and video games ( Figure 3). The indices to assess the models turned out to be of very good fit: CFI and TLI > 0.95, RMSEA < 0.06, SRMR < 0.05.

The structural regression coefficients show that although the latent variables distress and anxiety due to COVID-19 have direct and significant effects on each of the digital addictions, the impact on the criterion variables is varied. Distress impacts more strongly on internet addiction (β = 0.31, p < 0.001) while anxiety affects more strongly on smartphone addiction (β = 0.33, p < 0.001). The explained variances of the combined effects of the two exogenous latent variables are quite high on smartphone addiction (R ^2^ = 25%) and internet (R ^2^ = 19%) and to a lesser but equally significant extent on video games (R ^2^ = 6%).

## Discussion

Even though technologies such as internet, smartphone and videogames can offer benefits for people's lives if their use is controlled ( Capa-Luque et al., 2022; Ime et al., 2024), nevertheless, it should not be left aside the problems that could be generated in psychological and physical health when there is an problematic or addictive usage ( Peng et al., 2022; Ran et al., 2022). In this regard, the findings of the present study show that the two latent and exogenous variables (stress and anxiety due to COVID-19) have direct effects on digital addictions, while the exogenous variables present a positive covariance as expected by theory and it is concordant with what has been found by Li et al. (2024).

The general model explains 22%, but in the specific models the explained variance of the exogenous variables on smartphone addiction (25%), internet (19%) and video game addiction (6%) were varied. The internal validity of all models was supported by goodness-of-fit indices that were found to be very satisfactory (CFI and TLI > 0.95, RMSEA and SRMR ≤ 0.06).

The data support the proposed hypothesis that some personal factors such as anxiety and distress can also promote or hinder positive adaptation of individuals ( Hébert et al., 2022). In the pandemic context, students found an escape mechanism or refuge in the use of social networks through the Internet, smartphones and video games ( Ime et al., 2024; Capa-Luque et al., 2023), with the risk that their uncontrolled use becomes problematic or addictive ( Jalal et al., 2022; Sixto-Costoya et al., 2021). As this fact falls outside healthy parameters, it demands urgent attention to mitigate the negative impacts.

An alternative proposed by various researchers in the field of addictions to reduce the problematic use of interactive technologies would consist of strengthening awareness, self-control, skills and knowledge for a responsible use of digital resources ( Chung et al., 2019; Greenfield, 2018). Another alternative approach would involve exposing students to promotional educational programs of skills and strategies for the management of negative emotions (distress and anxiety) to help the problematic or addicted user to better understand and manage their emotional functioning, based on the premise that resorting to the use of digital technologies is an inadequate coping mechanism to psychological and physical discomfort generated by distress and anxiety would consist, as proposed by Taquet et al. (2017).

With respect to the psychological distress experienced in the context of the COVID-19 pandemic, the results show that the great majority of students presented distress (around 80%) and anxiety (70%) between moderate and high levels; proportionally, for every male, two females were found with high levels of distress and anxiety.

In this sense, the findings of the present study definitively confirm what was pointed out by Deng et al. (2021) and Saladino et al. (2020), regarding the fact that the university population is considered a risk group for developing stress and anxiety. It is evident that, in the pandemic context, due to the implications of the state of emergency and virtual education, university students were more vulnerable in their physical and psychological health and quality of life ( Parvar et al., 2022; Bazán-Ramírez et al., 2022).

Regarding the use of digital technologies such as smartphones and internet, about 40% of students were found in the category of problematic or clinically significant use with risk of addiction, affecting males and females equally. This category of problematic use was established for a cut-off point of 95 to 99 percentiles, and this would imply that students located in this category are not people who use these digital devices too much due to academic or work activities, but are very dependent users of these technologies. With respect to video games in the category of problematic use, the difference between males (18.8%) and females (2.7%) is quite marked. These results are consistent with several studies that report the increase in excessive use of digital connections; as referred by Meng et al. (2022), because they found a prevalence of 27% for addiction to cell phones, 8.23% to the internet and 6.04% to video games. Similarly, Sandoval et al. (2020) found the presence of internet addiction (31%) in university students who failed undergraduate courses and greater adherence to video games in men. Along the same lines, Ruiz-Palmero et al. (2021) found risk and abuse of video games and smartphones in university students.

The most important limitation of the study is its external validity due to the type of non-probabilistic sampling used, in this sense the possibility of generalization to other contexts should be carried out with caution; however, despite the limitation, the present study is considered important in view of the existing knowledge gap in relating the variables studied through explanatory models such as those addressed in the present study. That is, we consider that the findings obtained may be useful for mental health professionals to strengthen or expand knowledge, as well as to design programs aimed at facilitating effective management of negative emotions such as distress and anxiety, and to prevent or intervene early in behavioral problems related to digital addictions.

On the other hand, the model assumed in the study corresponds to structural regressions of latent variables of structural equation modeling (SEM), a strategy that allows the evaluation of weak causal relationships ( Keith, 2019), because direct causality corresponds to experimental designs, in the SEM causal relationships are given by the fulfillment of three conditions ( Byrne, 2010; Keith, 2019): 1. Existence of functional relationship between variables, 2. The cause precedes the effect in time (in a real or logical way) and the relationship must not be spurious. 3. The relationship must not be spurious. Therefore, despite the possible limitations of a cross-sectional study, the research satisfactorily fulfills the general objective of science of seeking models and theories to explain important facts of reality, which in the specific case of the present study consists of offering an explanation where the structural relationships between the constructs (latent variables) can imply causality (albeit weak, but causality) and covariances simultaneously.

It is also suggested to the agents of change and especially to the university authorities to promote the design and implementation of intervention programs aimed at reducing the problematic use of digital devices. It is urgent to raise awareness with timely information and expose to preventive programs for students to use interactive technologies such as smartphones, internet and video games to benefit from their responsible use and a means of social recreation and not a risk factor for the health of students.

## Conclusions

The results of the proposed structural regression models allow us to argue that stress and anxiety due to COVID-19 favor the abuse of technological devices and the increase of digital addictions in university students, thus affecting not only their physical and psychological health but also their academic and professional training.

The problematic use or addiction to the smartphone and internet affects a significantly larger group of students (about 40%) compared to addiction to video games that affects men (20%) and women (3%) differentially.

With respect to stress, 8 out of 10 students present problems of consideration in the moderate and high categories. About 70% of the students present anxiety due to COVID-19 between moderate and severe levels. In addition, proportionally for every male there are two females with high levels of distress and anxiety.

### Ethical approval and consent

The study was conducted in accordance with the Declaration of Helsinki and was approved by the Research Projects Office of the Central Institute of Research Management of the Vice Rectorate of Research, ratified the approval by the Faculty of Psychology of the Universidad Nacional Federico Villarreal by means of the Dean's Resolution N° 2076-2022-D-FAPS-UNFV dated March 30, 2022.

An informed consent form was designed and presented in the first section of the Google Form. Only students who read the consent form and agreed to participate in the research were invited to answer the instruments online. Participants were also informed that they were free to withdraw and stop filling out the instruments at any time by closing the virtual form without saving any information.

During the year 2022 classes in Peruvian universities were still virtual due to the COVID-19 pandemic, which is why obtaining written informed consent was sent to students' emails or WhatsApp accounts. Along with the consent letter, the link to access the google form was sent. Consent that was also submitted within the google form. Both the informed consent and the google form are accessible at Data Availability statement ( Capa, 2024).

This informed consent form was submitted as an annex together with the research project to the evaluating entity (Research Projects Office). The research project as well as its annexes (including the letter of consent used) complied with the approval report of the ethics committee of the Faculty of Psychology of the Federico Villarreal National University.

## Data Availability

Harvard Dataverse: Impact of distress and anxiety due to COVID-19 on digital addictions in university students in the third wave period. https://doi.org/10.7910/DVN/UHDRCW ( Capa, 2024) This project contains the following underlying data:
- Database research (Data_INV22_English.sav) Data are available under the terms of the License/Data Use Agreement PUBLIC DOMAIN (CC0 1.0). Harvard Dataverse: Impact of distress and anxiety due to COVID-19 on digital addictions in university students in the third wave period. https://doi.org/10.7910/DVN/UHDRCW ( Capa, 2024) This project contains the following underlying data:
- Psychometric properties of measuring instruments (Psychometric Properties.pdf ) - Annexes – Instruments.docx Data are available under the terms of the License/Data Use Agreement PUBLIC DOMAIN (CC0 1.0).
