# Supplementary material for: Impact of distress and anxiety due to COVID-19 on digital addictions in university students in the third wave period 
Source: F1000Res. 2025 Jan 27;13:1010. Originally published 2024 Sep 5. [Version 2] doi: 10.12688/f1000research.154696.2 (PMC11621610; doi:10.12688/f1000research.154696.2)
Supplement: Peer review history

# Reviewer report: Reviewer response for version 2

**DOI:** 10.5256/f1000research.176293.r362844

**Published:** 2026-01-02

**Authors:** Octaviano Garcia Robelo

**Affiliations:** 1 Universidad Autonoma del Estado de Hidalgo, Pachuca, Hidalgo, Mexico

The article demonstrates sufficient theoretical and methodological rigor and foundation.

 The bibliographic references are mostly from the last five years, are sufficient, and are correctly cited.

 The results support the importance of using technological devices and applications to strengthen the mental health of the medical students who participated in this research.

 There is sufficient methodological rigor, and the statistical analyses are valid.

 The article is important because it demonstrates that it is useful for students to use technological applications to strengthen and care for their mental health.

Is the work clearly and accurately presented and does it cite the current literature?

Yes

If applicable, is the statistical analysis and its interpretation appropriate?

Yes

Are all the source data underlying the results available to ensure full reproducibility?

Yes

Is the study design appropriate and is the work technically sound?

Yes

Are the conclusions drawn adequately supported by the results?

Yes

Are sufficient details of methods and analysis provided to allow replication by others?

Yes

Reviewer Expertise:

Education and psychology in higher education

I confirm that I have read this submission and believe that I have an appropriate level of expertise to confirm that it is of an acceptable scientific standard.

# Reviewer report: Reviewer response for version 1

**DOI:** 10.5256/f1000research.169754.r334071

**Published:** 2024-12-12

**Authors:** Octaviano Garcia Robelo

**Affiliations:** 1 Universidad Autonoma del Estado de Hidalgo, Pachuca, Hidalgo, Mexico

The article can be approved: No minor changes are required. The article analyzes and statistically explains the effects of anxiety and stress factors on digital addictions in university students. Its theoretical framework helps to theoretically substantiate the existence of stress and anxiety in more than a quarter and about half young college students in various countries around the world, which are shown and substantiated in their research reports. Also, it presents and details the digital additions, mainly cell phone and video games, in young people, which increased as a result of the Pandemic COVID_19. The methodology for both the data collection and the use of structural equations, allows confirming and explaining with precision the statistically significant influence of the two models shown. This methodology can be replicated for other investigations. The results contribute to substantiate the consequences in mental health brought about by the COVID_19 pandemic in university students in Peru. This phenomenon, which undoubtedly occurred and occurs in other countries of the world, should be addressed and investigated for the prevention of major consequences, such as suicide, low performance and dropping out of their studies. The references are updated and totally related to her research topic.

Is the work clearly and accurately presented and does it cite the current literature?

Yes

If applicable, is the statistical analysis and its interpretation appropriate?

Yes

Are all the source data underlying the results available to ensure full reproducibility?

Yes

Is the study design appropriate and is the work technically sound?

Yes

Are the conclusions drawn adequately supported by the results?

Yes

Are sufficient details of methods and analysis provided to allow replication by others?

Yes

Reviewer Expertise:

Education and psychology in higher education

I confirm that I have read this submission and believe that I have an appropriate level of expertise to confirm that it is of an acceptable scientific standard.

# Response

**Published:** 2025-01-17

**Authors:** ALDO BAZAN-RAMIREZ

**Affiliations:** Escuela de Psicología, Universidad Nacional José María Arguedas, Andahuaylas, APURÍMAC, Peru

**Dear Reviewer.**

**Your comments have helped improve our manuscript. I appreciate the time and interest that you have focused on reviewing and giving feedback on our work**

 One of our variables is distress and not stress, as can be seen in the title and throughout the paper.

 A correction has been made in the abstract, replacing a drafting error in the name of an instrument (COVID-19 worry scale) with the correct name (anxiety scale by COVID-19). The naming of the variables has been standardized throughout the article.

 Since we have used the structural equation modeling strategy, the relationship between the variables is expressed in terms of impact or direct effects of the exogenous variables on the endogenous or dependent variable.

 The variables distress and anxiety due to COVID-19 are unidimensional variables (no dimensions), and their description is shown in Table 1. The digital addictions variable is described by its dimensions (Smartphone, Internet and Video game) as shown in Table 2.

# Reviewer report: Reviewer response for version 1

**DOI:** 10.5256/f1000research.169754.r334066

**Published:** 2024-12-05

**Authors:** Georgina Eugenia Bazán Riverón

**Affiliations:** 1 Facultad de Estudios Superiore Iztacala, Universidad Nacional Autónoma De México, Mexico City, Mexico

The article meets the requirements. I only suggest a few recommendations:

 - The title refers to stress and anxiety. In the summary and in the method section it names another variable (worry) and it does not speak of anxiety, but rather worry. They are not the same. It is suggested that variables are always named the same way.

 - It seems to me that the problem statement can be more specific, detailing the relationship between variables.

 - In the results, a detailed description of the dimensions of the instruments would help to a deeper understanding.

 - Review punctuation and spaces in the text

Is the work clearly and accurately presented and does it cite the current literature?

Partly

If applicable, is the statistical analysis and its interpretation appropriate?

Yes

Are all the source data underlying the results available to ensure full reproducibility?

Yes

Is the study design appropriate and is the work technically sound?

Yes

Are the conclusions drawn adequately supported by the results?

Yes

Are sufficient details of methods and analysis provided to allow replication by others?

Yes

Reviewer Expertise:

NA

I confirm that I have read this submission and believe that I have an appropriate level of expertise to confirm that it is of an acceptable scientific standard.

# Reviewer report: Reviewer response for version 1

**DOI:** 10.5256/f1000research.169754.r322445

**Published:** 2024-09-12

**Authors:** Africa Borges, Leire Aperribai

**Affiliations:** 1 Department of Clinical Psychology, Psychobiology and Methodology, University of La Laguna, La Laguna, Spain; 2 Universidad del Pais Vasco, Bilbao, Basque Country, Spain

The article proposed for indexing has, in my view, sufficient value for this purpose. On the one hand, the subject is of great relevance, since the pandemic has been a turning point in life, both at the level of the various strata of society and from an international perspective. It has been a hard experience, but it is important to develop what we have been able to learn from it. In this sense, this study provides an important approach, relating additions that may have developed during the pandemic. The thoroughness of the analysis leaves no doubt as to its relevance.

Is the work clearly and accurately presented and does it cite the current literature?

Yes

If applicable, is the statistical analysis and its interpretation appropriate?

Yes

Are all the source data underlying the results available to ensure full reproducibility?

Yes

Is the study design appropriate and is the work technically sound?

Yes

Are the conclusions drawn adequately supported by the results?

Yes

Are sufficient details of methods and analysis provided to allow replication by others?

Yes

Reviewer Expertise:

Methodology of Behaioral Sciences

We confirm that we have read this submission and believe that we have an appropriate level of expertise to confirm that it is of an acceptable scientific standard.
